# Life-threatening emphysematous liver abscess associated with poorly controlled diabetes mellitus: a case report

**DOI:** 10.1186/s13104-017-2445-8

**Published:** 2017-03-06

**Authors:** Yuichi Takano, Masafumi Hayashi, Fumitaka Niiya, Toru Nakanishi, Shotaro Hanamura, Kunio Asonuma, Eiichi Yamamura, Kuniyo Gomi, Yuichiro Kuroki, Naotaka Maruoka, Kazuaki Inoue, Masatsugu Nagahama

**Affiliations:** Division of Gastroenterology, Department of Internal Medicine, Showa University Fujigaoka Hospital, 1-30 Fujigaoka, Aoba-ku, Yokohama, Kanagawa 227-8501 Japan

**Keywords:** Emphysematous liver abscess, Gas-forming pyogenic liver abscesses, Diabetes mellitus

## Abstract

**Background:**

Emphysematous liver abscesses are defined as liver abscesses accompanied by gas formation. The fatality rate is extremely high at 27%, necessitating prompt intensive care.

**Case presentation:**

The patient was a 69-year-old Japanese man with type 2 diabetes. He visited the emergency outpatient department for fever and general malaise that had been ongoing for 2 weeks. Abdominal computed tomography revealed an abscess 5 cm in diameter accompanied by gas formation in the right hepatic lobe. Markedly impaired glucose tolerance was observed with a blood sugar level of 571 mg/dL and a glycated hemoglobin level of 14.6%. The patient underwent emergency percutaneous abscess drainage, and intensive care was subsequently initiated. *Klebsiella pneumoniae* was detected in both the abscess cavity and blood cultures. The drain was removed 3 weeks later, and the patient was discharged.

**Conclusion:**

Emphysematous liver abscesses are often observed in patients with poorly controlled diabetes, and the fatality rate is extremely high. Fever and malaise occasionally mask life-threatening infections in diabetic patients, necessitating careful examination.

## Background

Emphysematous liver abscesses are also referred to as gas-forming pyogenic liver abscesses and are defined as liver abscesses accompanied by gas formation [1–3]. Emphysematous liver abscesses account for 6–24% of bacterial liver abscesses and are often observed in patients with poorly controlled diabetes, making them susceptible to sepsis and rupture [2, 4, 5]. The fatality rate is extremely high at 27%, necessitating prompt intensive care [2]. We report a case of an emphysematous liver abscess that occurred in a patient with poorly controlled diabetes.

## Case presentation

The patient was a 69-year-old Japanese man with a history of type 2 diabetes since 7 years ago. He had been taking oral medication for it but had stopped treatment on his own accord 3 years earlier. He had no co-morbids of diabetes.

He visited the emergency department for fever and general malaise that had been ongoing for 2 weeks. His vital signs were a body temperature of 38.2 °C, blood pressure of 144/60 mmHg, heart rate of 131 bpm, and saturation of peripheral oxygen (SpO_2_) of 95% (room air). While tenderness was observed in the right hypochondrium, no signs of peritoneal irritation were observed. Blood tests revealed markedly elevated inflammatory response with a white blood cell count of 16,400/μL and C-reactive protein level of 26.5 mg/dL. His aspartate transaminase (AST) level was 371 U/L, alanine aminotransferase (ALT) level was 331 U/L, alkaline phosphatase (ALP) level was 675 U/L, and gamma-glutamyl transpeptidase (γ-GTP) level was 197 mg/dL, indicating liver dysfunction. Procalcitonin (PCT) was high at 26.23 ng/mL, suggesting sepsis. In addition, markedly impaired glucose tolerance was noted with a blood sugar level of 571 mg/dL and glycated hemoglobin (HbA1c) level of 14.6%. The patient tested negative for urine ketones and had an arterial blood pH of 7.36, indicating no acidosis.

**Fig. 1:**
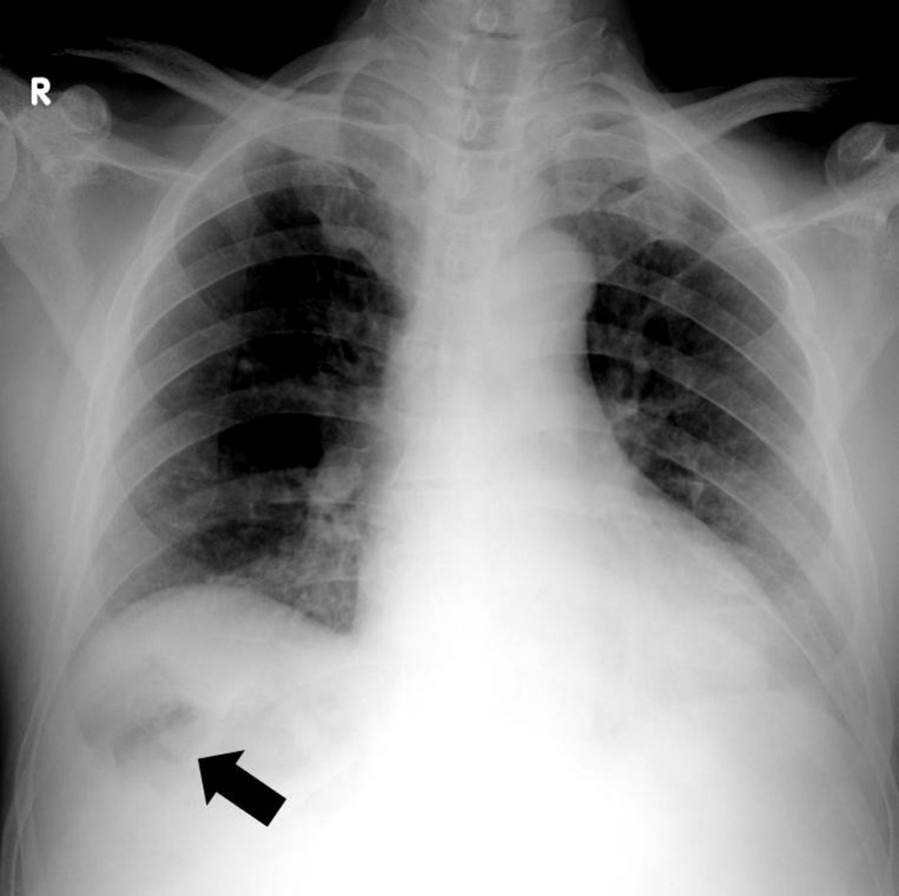
Gas was observed in the right subphrenic space on simple abdominal radiography (*arrow*)

**Fig. 2:**
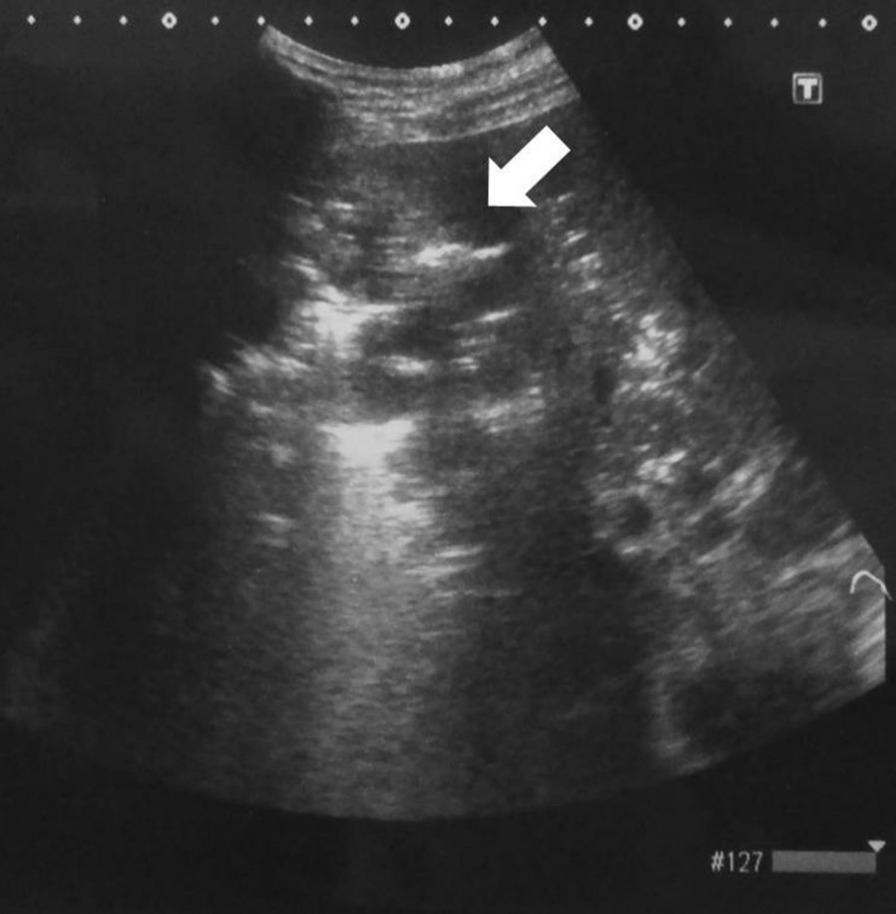
A hypoechoic lesion with indistinct boundaries was observed in the right hepatic lobe on abdominal ultrasound, and several hyperechoic findings suggestive of air were observed within this lesion (*arrow*)

**Fig. 3:**
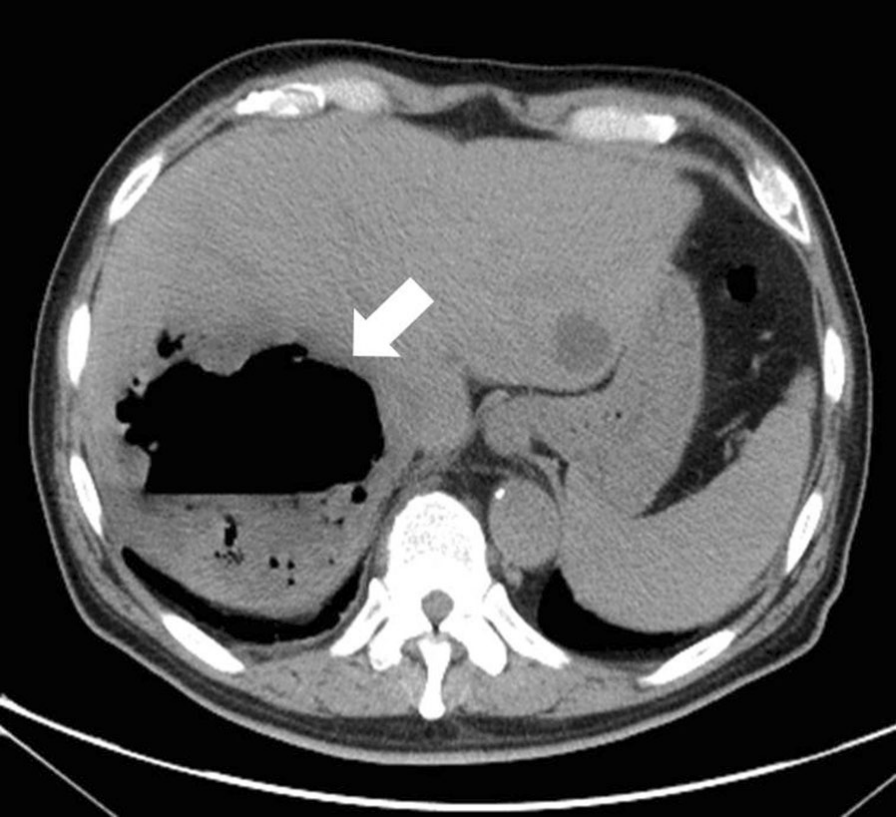
An abscess 5 cm in diameter was observed in the right hepatic lobe on abdominal computed tomography, within which gas-related fluid formation was detected (*arrow*). A cyst was observed in the left lobe

**Fig. 4:**
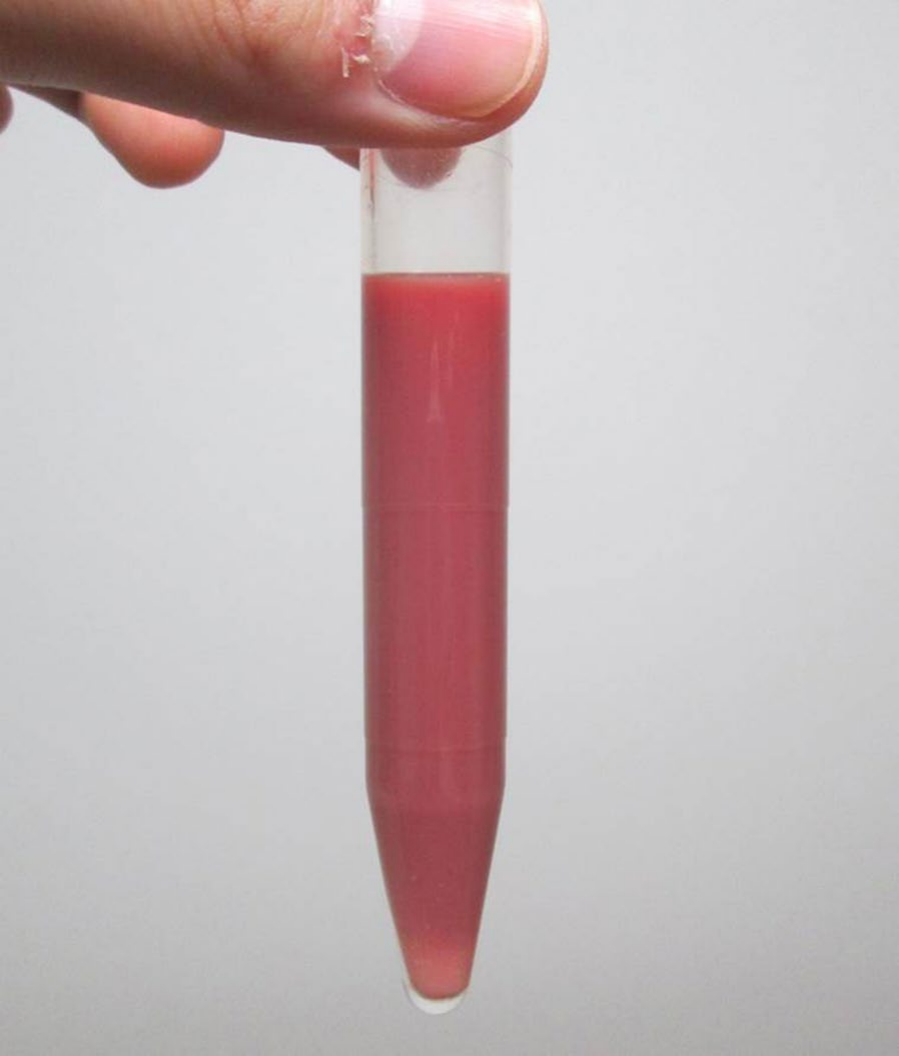
Effluent drained from the abscess. *Reddish-brown* purulent effusion was observed

Simple abdominal radiography revealed gas in the right subphrenic space (Fig. 1). A hypoechoic lesion with indistinct boundaries was also noted in the right hepatic lobe on abdominal ultrasound, and a number of hyperechoic findings suggestive of air were observed within this lesion (Fig. 2). Abdominal computed tomography (CT) revealed an abscess 5 cm in diameter in the right hepatic lobe, within which gas-related fluid formation was observed (Fig. 3). The patient underwent emergency percutaneous abscess drainage and placement of an 8-Fr tube. The aspirate was reddish-brown and purulent, and *Klebsiella pneumoniae* was detected in cultures (Fig. 4). The same bacterium was also detected in two blood cultures. The sensitivity is presented in Table 1. A pathological examination of the drained effluent was also conducted, revealing no malignant findings.

**Table 1:** The sensitivity of *Klebsiella pneumoniae*

	MIC	Sensitivity
Ampicillin	>16	R
Levofloxacin	<1	S
Ceftazidime	<2	S
Tazobactam/piperacillin	<8	S
Amikacin	<8	S
Fosfomycin	<4	S
Sulbactam/ampicillin	<8	S
Gentamicin	<1	S
Ceftriaxone	<1	S
Meropenem	<1	S
Cefmetazole	<2	S

The patient was admitted to the intensive care unit and started on antibiotics (meropenem at 3 g/day) and glucose control by continuous intravenous infusion of insulin. Four days later, the patient was transferred to the general ward. Based on culture sensitivity, the antibiotic was changed to ceftriaxone at 4 g/day, which was continued for 2 weeks. Upper and lower endoscopy revealed no evident abnormalities, and magnetic resonance cholangiopancreatography (MRCP) revealed no gallstones or biliary tract tumors. The patient tested negative for human immunodeficiency virus (HIV) antibodies, and, 3 weeks later, the drainage tube was removed and the patient was discharged without sequela.

## Discussion

Emphysematous liver abscesses were defined as liver abscesses accompanied by gas formation and first reported by Smith in 1944 [6]. Emphysematous liver abscesses account for 6–24% of bacterial liver abscesses and typically accompany poorly- controlled (HbA1c > 8.0%) diabetes at a high rate of 76–86% [1, 2, 4, 5, 7]. The most common causative bacterium is *K. pneumoniae*, accounting for approximately 70% of cases [3]. This disease is rare in the West, but common in Asia, particularly Taiwan [5].

Emphysematous liver abscesses are prone to septic shock and rupture. Chou et al. [2] observed septic shock in 32% of emphysematous liver abscesses that they examined and reported non gas-forming liver abscesses in 11% of cases. Rupture is also prone to occur as a result of strong tissue damage and rises in internal pressure due to gas formation. The fatality rate (27–30%) is higher than that of non gas-forming liver abscess (2–12%) [2, 8, 9]. Abdominal ultrasound, simple abdominal radiography, and other imaging techniques are useful for diagnosis, but CT is the best method for sensitive detection of gas within abscesses.

Hyperglycemia is considered to be deeply involved in gas formation [10]. Hyperglycemia is reported to promote gas production by further enhancing the glucose metabolism of microorganisms. Lee et al. [5] analyzed the gas within liver abscesses and found that it contained hydrogen gas; this led to their speculation that mixed acid fermentation of glucose is involved in gas formation.

In addition to antibiotics and glucose control, percutaneous abscess drainage is often required for treatment. Lee et al. [5] examined 22 cases of emphysematous liver abscess and reported percutaneous transhepatic abscess drainage in 19 cases and surgery in one case. Drainage is particularly recommended in cases of large abscesses exceeding 5 cm in diameter [11]. In cases of rupture and peritonitis, surgery should be considered.

Our case was instructive because the chief complaints were fever and general malaise, without abdominal symptoms. Hagiya et al. [1] similarly reported two cases of emphysematous liver abscess without any abdominal symptoms. The reason for this is likely diabetic neuropathy or a similar condition. Prolonged fever and malaise in patients with poorly controlled diabetes could mask a life-threatening infection and thus require careful attention.

## Conclusion

Emphysematous liver abscesses are often observed in patients with poorly controlled diabetes, and the fatality rate is extremely high. Fever and malaise occasionally mask life-threatening infections in diabetic patients, necessitating careful examination.

## Abbreviations

SpO2: saturation of peripheral oxygen
AST: aspartate transaminase
ALT: alanine aminotransferase
ALP: alkaline phosphatase
γ-GTP: gamma-glutamyl transpeptidase
PCT: procalcitonin
HbA1c: glycated hemoglobin
CT: computed tomography
MRCP: magnetic resonance cholangiopancreatography
HIV: human immunodeficiency virus
